# Self-Medication With Over-the-counter Medicines Among the Working Age Population in Metropolitan Areas of Thailand

**DOI:** 10.3389/fphar.2021.726643

**Published:** 2021-08-11

**Authors:** Sineenart Chautrakarn, Waraporn Khumros, Phanupong Phutrakool

**Affiliations:** ^1^ Faculty of Public Health, Chiang Mai University, Chiang Mai, Thailand; ^2^ Faculty of Nursing, Siam University, Bangkok, Thailand; ^3^ Faculty of Medicine, Chulalongkorn University, Bangkok, Thailand

**Keywords:** self-medication, antibiotics stewardship, metropolitan, thailand, working age population, over-the-counter medicines

## Abstract

**Background and Objectives:** Self-medication with over-the-counter (OTC) medicines is becoming an increasingly popular practice around the world. The global prevalence rate of self-medication ranges from 11.2% to 93.7%, depending on the target population and country. However, there is a lack of data on the prevalence and practices of self-medication among the working-age population, particularly in Thailand metropolitan areas. The current study describes the prevalence of self-medication practices, adverse drug reactions and severity, reasons for self-medication, and basic medication knowledge among people of working age in metropolitan areas in Thailand.

**Methods:** We conducted an online cross-sectional study between December 2020 and January 2021. Descriptive statistics were used to analyze self-medication data. A chi-square test was used to assess the association between self-medication and sociodemographic characteristics.

**Results:** This study found high prevalence of self-medication among the working-age population in metropolitan areas of Thailand (88.2%). The most commonly used drug groups were NSAIDs (34.8%) and antibiotics (30.2%). Minor illness and easy access to pharmacies were the most common reasons for self-medication. Almost half of the participants&apos; illnesses (42.6%) for which they self-medicated were not always completely cured, necessitating treatment at a hospital or clinic. Although only a small number of participants (ranged from 0.6 to 6.6%) experienced adverse drug reactions as a result of self-medication, some had severe symptoms that disrupted their daily lives or required hospitalization. In terms of basic medication knowledge, we discovered that study participants misunderstood some antibiotic drug concepts.

**Conclusions:** According to the study findings, it is recommended that more information about the risks of self-medication, drug adverse reactions, antibiotic stewardship, more supervision of the prohibition of over-the-counter drugs and selling practices, and adequate facilities for peoples access to medical services be provided at the policy level.

## Introduction

Over-the-counter (OTC) medicines, also known as nonprescription medicines, refer to medications that can be purchased without a prescription and are safe and effective when used according to the directions on the label, and as directed by a health care professional (Food and Drug Admini, 2018). Self-medication is becoming increasingly popular around the world. According to studies, the global prevalence of self-medication ranges from 11.2 to 93.7%, depending on the target population and country (Balbuena et al., 2009; Kasulkar and Gupta, 2015; Arrais et al., 2016; Håkonsen et al., 2016; Prado et al., 2016; Gama and Secoli, 2017; Helal and Abou-ElWafa, 2017; Abdi et al., 2018; Kassie et al., 2018; Lei et al., 2018; Tesfamariam et al., 2019). This means a large proportion of the world’s population uses drugs without first consulting a doctor or healthcare professional.

Individuals and the health-care sector can both benefit from good self-medication practices. For example, decreasing absenteeism from work due to minor illnesses, saving time and money spent on doctor visits, and reducing (or at least optimizing) the burden on governments due to health expenditure associated with the treatment of minor health conditions (Hughes et al., 2001; Ruiz, 2010; Sarahroodi, 2012). According to recent studies, among the most common reasons for people to self-medicate are the convenience of going to a pharmacy rather than seeing a doctor and avoiding the need to go to a hospital for treatment (Kassie et al., 2018; Lei et al., 2018). However, inappropriate self-medication with OTC drugs can lead to drug-related problems and have serious consequences (including death) (Ruiz, 2010; Eickhoff et al., 2012; Sarahroodi, 2012; Asseray et al., 2013; Oliveira et al., 2014). Globally, the increasing rate of inappropriate self-medication is becoming a public health concern, particularly antimicrobial resistance caused by inappropriate antibiotic use (Watkins and Bonomo, 2016).

Thailand’s working population is defined as those aged 15 to 59. The majority of this group resides in the country’s metropolitan areas. The country’s cities are generally densely populated. Within this dense, urban society, people move in a hurry, competing against time, and their commutes are lengthened by traffic congestion. As a result, when illness strikes, some people opt for alternative methods of treatment rather than going to the doctor for medical treatment.

As of March 2017, Thailand had 22,459 drug stores, with Bangkok accounting for one-third of them. This number is expected to rise year after year, particularly in the metropolitan and urban areas (Tunpaiboon, 2018). Given the country’s growing number of pharmacy stores as well as the lack of data on the prevalence and practices of self-medication among the working-age population, particularly in Thailand’s metropolitan areas, this study sought to fill the gap in the extant literature. The current study was designed to describe the prevalence of self-medication practices, adverse drug reactions and their severity, reasons for self-medication, and basic medication knowledge among working-age people. The findings are expected to contribute to policy and regulatory changes aimed at encouraging appropriate self-medication.

## Methods

### Study Design and Setting

This cross-sectional study was conducted online between December 2020 and January 2021 among working age people in Bangkok and Chiang Mai province, Thailand’s metropolitan areas.

### Participants

Participants in the study ranged in age from 15 to 59 years old and lived in Bangkok or Chaing Mai province. Medical doctors were excluded from the study because they are well-versed in treatment and have full authority to prescribe medications to themselves and others. Our sample size was calculated by estimating a finite population proportion based on the total number of people aged 15–59 years in Bangkok in 2017 (3,688,424 people) (Administrative Strategy D, 2017). We added a 30% non-response rate, yielding a final sample size of 397. However, to obtain more representative data, we decided to collect as much sample data as possible during the study period. In this study, we used convenience sampling and snowball sampling to collect up to 786 samples.

### Data Collection

The research information sheet and informed consent form were made available to study subjects online, using responsive web applications. The study subjects were initially screened for inclusion criteria including age, place of residence, and occupation. To participate in the study, they were asked to sign an informed consent form by clicking on it, after which the questionnaire would appear on the screen.

### Questionnaire Development and Testing

Investigators created a questionnaire based on a review of the literature and real-world situations in the country (Balbuena et al., 2009; Ruiz, 2010; Eickhoff et al., 2012; Asseray et al., 2013; Arrais et al., 2016; Håkonsen et al., 2016; Aziz et al., 2018; Kassie et al., 2018; Lei et al., 2018; Shafie et al., 2018). To collect data, a self-administered online questionnaire with five sections was used. The first section of the questionnaire related to participants sociodemographic information, such as sex, age, marital status, level of graduation, field of study, occupation, monthly income, underlying history of drug allergy, and health insurance.

The second section of the questionnaire asked about self-medication practices during the previous year, including frequency and patterns of self-medication, types of medicines used, outcomes, and pharmacy-related experiences such as reasons for choosing a drug store, whether they met the pharmacist every time they purchased medicine from the drug store, and how frequently they received drug use advice from the seller. We were interested in some types of drugs that should be used under adequate supervision by healthcare professionals. These included NSAIDs, antibiotics, anti-allergic drugs, muscle relaxants, topical steroids, traditional herbal registration, eye drops or eye ointments, anti-migraine drugs, oral contraceptives or oral hormones, injection drugs, and isotretinoin). This section also asked the respondents if they had experienced any adverse drug reactions as a result of self-medication, such as palpitations, nausea/vomiting, dizziness, itching/rash, diarrhea, dyspnea, blurred vision, gastric upset caused by NSAIDs, and steroid acne or topical steroid addiction caused by topical steroids. Each symptom could be answered with a no, not sure, or yes. If participants said yes, they would be questioned about the severity of the event.

The third section of the questionnaire asked participants to express their agreement or disagreement on the reasons for self-medication using a five-point scale questionnaire, where 5 = strongly agree, 4 = agree, 3 = neutral, 2 = disagree, and 1 = strongly disagree. There were 14 items in this section.

The final section of the questionnaire included questions about basic medication knowledge with which most people should be familiar when practicing self-medication. There were 18 questions in total.

The questionnaire was reviewed and evaluated by three experts; one infectious diseases physician, one university lecturer with a pharmacy background, and one university lecturer with an epidemiology background, and their corrective comments were implemented. The questionnaire was given to 30 working age people to assess its reliability, and a Cronbach’s alpha test was performed, yielding a reliability index of 0.84.

### Statistical Analysis

The obtained data were analyzed by SPSS-22 software using descriptive statistics (frequency, mean, and standard deviation). A chi-square test was used to compare the sociodemographic characteristics of self-medication and non-self-medication groups. The level of significance was set at *p* < 0.05.

## Results

### Sociodemographic Characteristics of the Respondents

In this study, 801 participants were approached, of whom 786 provided completed data for analysis (98.1%). Seventy two percent (566) of the participants were female. The mean age of the respondents was 38.06 ± 8.11; 471 (59.9%) were single; and 684 (87.0%) were under health insurance coverage. In terms of education level, 351 (44.7%) participants held a bachelor’s degree and 325 (41.3%) held a master’s degree. In terms of fields of study, 213 (27.1%) of the participants were in health sciences and 179 (22.8%) of them studied in the science and technology fields (Table 1).

**TABLE 1:** Sociodemographic characteristics of respondents by self-medication (*n* = 786).

Variables	Self-medication (*n* = 693)	Non-self-medication (*n* = 93)	Total (*n* = 786)	*p*-value
**Sex**				0.003^a^
Male	155 (81.2%)	36 (18.8%)	191 (24.3%)	
Female	512 (90.5%)	54 (9.5%)	566 (72.0%)	
LGBTQ	26 (89.7%)	3 (10.3%)	29 (3.7.0%)	
**Marital status**				0.233
Single	408 (86.6%)	63 (13.4%)	471 (59.9%)	
Married	269 (90.3%)	29 (9.7%)	298 (37.9%)	
Divorced/Widowed	16 (94.1%)	1 (5.9%)	17 (2.2%)	
**Level of graduation**				0.012^a^
Less than bachelor degree	17 (89.5%)	2 (10.5%)	19 (2.4%)	
Bachelor degree	296 (84.3%)	55 (15.7%)	351 (44.7%)	
Master degree	293 (90.2%)	32 (9.8%)	325 (41.3%)	
Doctoral degree	87 (95.6%)	4 (4.4%)	91 (11.6%)	
**Field of study**				0.971
Heath sciences	189 (88.7%)	24 (11.3%)	213 (27.2%)	
Humanities/social Sciences	138 (88.5%)	18 (11.5%)	156 (19.8%)	
Science and technology	158 (88.3%)	21 (11.7%)	179 (22.8%)	
Finance and accounting	93 (86.1%)	15 (13.9%)	108 (13.7%)	
Others (not specified)	115 (88.5%)	15 (11.5%)	130 (16.5%)	
**Occupation**				0.176
Unemployed	23 (95.8%)	1 (4.2%)	24 (3.0%)	
Daily workers	35 (92.1%)	3 (7.9%)	38 (4.8%)	
Self-employed	123 (90.4%)	13 (9.6%)	136 (17.3%)	
Government official /Government employee /State enterprise employee	227 (89.7%)	26 (10.3%)	253 (32.3%)	
Office workers	254 (84.4%)	47 (15.6%)	301 (38.3%)	
Freelance	31 (91.2%)	3 (8.8%)	34 (4.3%)	
**Being a health care provider**				0.918
No	525 (88.2%)	70 (11.8%)	595 (75.7%)	
Yes	168 (88.0%)	23 (12.0%)	191 (24.3%)	
**Monthly income (THB)**				0.313
< 10,000	39 (86.7%)	6 (13.3%)	45 (5.7%)	
10,000 – 30,000	179 (85.6%)	30 (14.4%)	209 (26.6%)	
30,001 – 50,000	207 (87.3%)	30 (12.7%)	237 (30.2%)	
50,001 up	268 (90.8%)	27 (9.2%)	295 (37.5%)	
**Underlying disease(s)**				0.003^a^
No	497 (86.1%)	80 (13.9%)	577 (73.4%)	
Yes	196 (93.8%)	13 (6.2%)	209 (26.6%)	
**Drug allergy history**				0.045^a^
No	559 (87.1%)	83 (12.9%)	642 (81.7%)	
Yes	134 (93.1%)	10 (6.9%)	144 (18.3%)	
**Health insurance**				<0.001^a^
No	101 (99.0%)	1 (1.0%)	102 (13.0%)	
Yes	592 (86.5%)	92 (13.5%)	684 (87.0%)	

a Significance level (THB: Thai Baht).

When the sociodemographic characteristics of the self-medication and non-self-medication groups were compared, statistically significant differences in sex, age, level of graduation, underlying disease(s), drug allergy history, and health insurance were found, as shown in Table 1.

### Prevalence of Self-Medication Practices

This study found that the overall prevalence of self-medication was 693 (88.2%) (95% CI: 85.6, 90.1), and was similar across fields of study, healthcare provider status, and monthly income, but varied by gender, marital status, level of graduation, occupation, underlying disease(s), drug allergy history, and health insurance (Table 1). It should be noted that the prevalence of self-medication was higher among those with a history of drug allergy than among those without a history of drug allergy.

### Self-Medication Practices

The majority of participants reported purchasing medications less than once a month (78.8%) or 1–2 times per month (18.8%). It should be noted that they chose which drugs to buy rather than allowing the seller to make drug recommendations (60.9 and 39.1%, respectively). After taking those medications, they reported that they recovered from 50.4% of their illnesses and 42.6% of their illnesses were frequently cured. For those who did not always recover from their illnesses, 76.2% sought treatment at a hospital or clinic, 19.8% allowed the illness to heal on its own, and 4.1 percent purchased additional medications. In this study, the most prevalent drug groups used were NSAIDs (34.8%), antibiotics (30.2%), and anti-allergic drugs (28.4%) (Table 2).

**TABLE 2:** Drug groups used for self-medication (*n* = 693).

Drug groups	n (%)
**Major drug class**	
NSAIDs	241 (34.8%)
Antibiotics	209 (30.2%)
Anti-allergic drugs	197 (28.4%)
Muscle relaxants	98 (14.1%)
Traditional Herbal Registration	57 (8.2%)
Anti-migraine drugs	32 (4.6%)
Oral contraceptives or oral hormones	28 (4.0%)
Isotretinoin	12 (1.7%)
**Drug used according to body organs**	
Topical steroids	62 (8.9%)
Eye drops or eye ointments	56 (8.1%)
**Other dosage form**	
Injection drugs	24 (3.5%)

In terms of pharmacy experiences, the most common reasons for choosing a drug store were the presence of a pharmacist (43.9%) and the store’s proximity to the home or office (42.7%). The majority of study participants reported that pharmacists are always or occasional sellers when they purchase medicines (45.2 and 37.7%, respectively). Only 2.9% had never met a pharmacist while purchasing medicine in a store, and 14.3% were unsure whether the seller was a pharmacist or not. In addition, 47.6% of study participants reported that they received drug use advice from sellers on a regular basis, while 47.2% received advice occasionally. Only 5.2% stated that they never got any advice from the seller when purchasing drugs in a store. Furthermore, 45.5% of participants reported regularly being asked about their history of drug allergies and/or pregnancy status, 41.0% were asked occasionally, and 13.6% were never asked about their history of drug allergies and/or pregnancy status by the seller.

### Adverse Drug Reactions

The results showed that a small number of participants experienced adverse drug reactions as a result of taking medications they purchased on their own (range from 0.6 to 6.6%). The most common adverse drug reaction was gastric upset caused by NSAIDs (6.6%), and the least common was blurred vision (0.6%). Most of those who experienced adverse drug reactions referred to their severity as minor or moderate. However, severe symptoms requiring a break or medical treatment were reported in those who experienced palpitations, nausea/vomiting, dizziness, itching/rash, diarrhea, dyspnea, blurred vision, gastric upset caused by NSAIDs, and steroid acne or topical steroid addiction caused by topical steroids (5.1, 9.1, 20.7, 17.9, 13.0, 25.0, 50.0, 19.6, and 14.3%, respectively). Furthermore, serious reactions requiring hospitalization were reported in those who experienced palpitations, itching/rash, and gastric upset caused by NSAIDs (5.1, 7.1 and 2.2%, respectively) (Table 3).

**TABLE 3:** Adverse drug reactions (*n* = 693).

Adverse drug reactions	n (%)	Adverse drug reactions	n (%)
**Gastric upset caused by NSAIDs**		**Dizziness**	
Never buy NSAIDs	354 (51.1%)	No	596 (86.0%)
No	249 (35.9%)	Not sure	68 (9.8%)
Not sure	44 (6.3%)	Yes	29 (4.2%)
Yes	46 (6.6%)	If yes, the severities were; (n = 29)	
If yes, the severities were; (n = 46)		Minor, unobtrusive to daily life	13 (44.8%)
Minor, unobtrusive to daily life	15 (32.6%)	Moderate, interfering with some aspects of daily life	10 (34.5%)
Moderate, interfering with some aspects of daily life	21 (45.7%)	Severe, causing significant disruption in daily life and necessitating a break or medical treatment	6 (20.7%)
Severe, causing significant disruption in daily life and necessitating a break or medical treatment	9 (19.6%)	Serious enough to necessitate hospitalization	0 (0.0%)
Serious enough to necessitate hospitalization	1 (2.2%)	**Itching/rash**	
**Palpitation**		No	614 (88.6%)
No	606 (87.4%)	Not sure	51 (7.4%)
Not sure	48 (6.9%)	Yes	28 (4.0%)
Yes	39 (5.6%)	If yes, the severities were; (n = 28)	
If yes, the severities were; (n = 39)		Minor, unobtrusive to daily life	14 (50.0%)
Minor, unobtrusive to daily life	23 (59.0%)	Moderate, interfering with some aspects of daily life	7 (25.0%)
Moderate, interfering with some aspects of daily life	12 (30.8%)	Severe, causing significant disruption in daily life and necessitating a break or medical treatment	5 (17.9%)
Severe, causing significant disruption in daily life and necessitating a break or medical treatment	2 (5.1%)	Serious enough to necessitate hospitalization	2 (7.1%)
Serious enough to necessitate hospitalization	2 (5.1%)	**Diarrhea**	
**Steroid acne or topical steroid addiction caused by topical steroids**		No	643 (92.8%)
Never buy topical steroids	357 (51.5%)	Not sure	27 (3.9%)
No	237 (34.2%)	Yes	23 (3.3%)
Not sure	64 (9.2%)	If yes, the severities were; (n = 23)	
Yes	35 (5.1%)	Minor, unobtrusive to daily life	14 (60.9%)
If yes, the severities were; (n = 35)		Moderate, interfering with some aspects of daily life	6 (26.1%)
Minor, unobtrusive to daily life	16 (45.7%)	Severe, causing significant disruption in daily life and necessitating a break or medical treatment	3 (13.0%)
Moderate, interfering with some aspects of daily life	14 (40.0%)	Serious enough to necessitate hospitalization	0 (0.0%)
Severe, causing significant disruption in daily life and necessitating a break or medical treatment	5 (14.3%)	**Dyspnea**	
Serious enough to necessitate hospitalization	0 (0.0%)	No	662 (95.5%)
**Nausea/vomiting**		Not sure	23 (3.3%)	
No	614 (88.6%)	Yes	8 (1.2%)
Not sure	46 (6.6%)	If yes, the severities were; (n = 8)	
Yes	33 (4.8%)	Minor, unobtrusive to daily life	3 (37.5%)
If yes, the severities were; (n = 33)		Moderate, interfering with some aspects of daily life	3 (37.5%)
Minor, unobtrusive to daily life	14 (42.4%)	Severe, causing significant disruption in daily life and necessitating a break or medical treatment	2 (25.0%)
Moderate, interfering with some aspects of daily life	16 (48.5%)	Serious enough to necessitate hospitalization	0 (0.0%)
Severe, causing significant disruption in daily life and necessitating a break or medical treatment	3 (9.1%)	**Blurred vision**	
Serious enough to necessitate hospitalization	0 (0.0%)	No	673 (97.1%)
		Not sure	16 (2.3%)
		Yes	4 (0.6%)
		If yes, the severities were; (n = 4)	
		Minor, unobtrusive to daily life	2 (50.0%)
		Moderate, interfering with some aspects of daily life	0 (0.0%)
		Severe, causing significant disruption in daily life and necessitating a break or medical treatment	2 (50.0%)
		Serious enough to necessitate hospitalization	0 (0.0%)

### Reasons for Self-Medication

**FIGURE 1:**
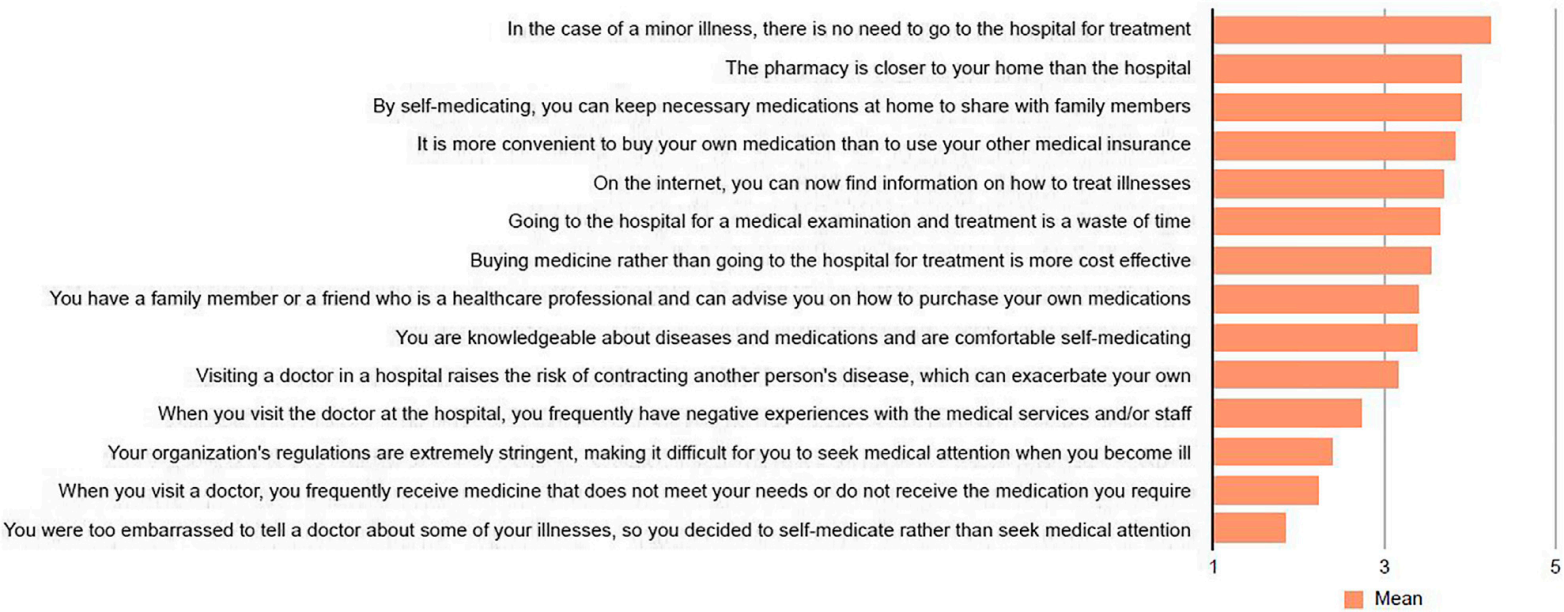
Reasons for self-medication, Self-medication with over-the-counter medicines among the working age population in metropolitan areas of Thailand, Thailand 2021.

In this study, we discovered a variety of reasons for self-medication (see Figure 1). Most of the participants believed there was no need to go to the hospital for treatment in the case of minor illness. Another reason is that a pharmacy was located closer to their homes than a hospital. They also agreed that self-medicating allowed them to keep the necessary medications at home to share with family members and that buying their own medication was more convenient than using their other medical insurance. Furthermore, they thought that they could find information on how to treat illnesses on the Internet.

### Basic Medication Knowledge

**FIGURE 2:**
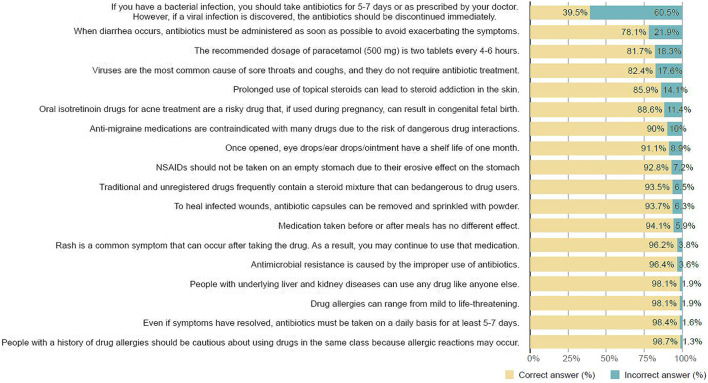
Basic medication knowledge, Self-medication with over-the-counter medicines among the working age population in metropolitan areas of Thailand, Thailand 2021.

The findings revealed that almost all participants correctly recognized the basic medication knowledge (Figure 2). However, we discovered that less than 80% of participants correctly determined the truthfulness of the following two statements: “When diarrhea occurs, antibiotics must be administered as soon as possible to avoid exacerbating the symptoms,” and “If you have a bacterial infection, you should take antibiotics for 5–7 days or as prescribed by your doctor; however, if you have a viral infection, you should stop taking antibiotics immediately” (78.1%, 39.5%, respectively). This meant that some or the majority of participants held misunderstandings about antibiotic use (Figure 2). We also asked participants about their knowledge of rational drug use and antibiotic stewardship and whether they had heard of those terms. The vast majority (74.3%) had. Surprisingly, a quarter of the participants knew nothing about rational drug use or antibiotic stewardship. The most common sources of information for participants who knew about rational drug use and antibiotic stewardship were hospital publicity boards and brochures (80.6%) and Facebook (53.2%). Participants received less information from drug stores and radios (30.5%, 27.6%, respectively).

## Discussion

The prevalence of self-medication varies by country and target population, and ranges from 11.2 to 93.7% (Balbuena et al., 2009; Kasulkar and Gupta, 2015; Arrais et al., 2016; Håkonsen et al., 2016; Prado et al., 2016; Gama and Secoli, 2017; Helal and Abou-ElWafa, 2017; Abdi et al., 2018; Kassie et al., 2018; Lei et al., 2018; Tesfamariam et al., 2019). In the present study, the prevalence of self-medication in the year prior to the study was 88.2%. The difference in self-medication rates could be attributed to differences in the study samples sociodemographic characteristics, research methodology, population and samples, data collection tools, and the operative definition of self-medication. We tend to think the high prevalence of self-medication is due to people with extensive knowledge of pharmaceutical products, such as doctors, nurses, pharmacists, and other healthcare professionals. According to recent studies, people with a background in health sciences have a high rate of self-medication (Woźniak-Holecka et al., 2012; Abdi et al., 2018). Surprisingly, the present study revealed that the prevalence of self-medication in the health sciences field was around 88%, the same rate as in the humanities and social sciences, and other fields (that were not specified in this study). Furthermore, this study found that the prevalence of self-medication was also high in the non-healthcare provider group (88.2%). This demonstrated that despite the fact people are not healthcare professionals, they have a tendency to self-medicate with over-the-counter medications. This phenomenon, however, may lead to drug-related problems if the self-medicators do not have accurate information about drug use or if pharmacists at drug stores do not provide adequate recommendations.

In the current study, NSAIDs (34.8%), antibiotics (30.2%), and anti-allergic drugs (28.4%) were the most frequently used medications. In various other studies conducted on self-medication, different drug groups for self-medication were reported. In this regard, the most prevalent medicine categories used included analgesics/NSAIDs (Woźniak-Holecka et al., 2012; Arrais et al., 2016; Fernando et al., 2017; Aziz et al., 2018; Shafie et al., 2018; Tesfamariam et al., 2019; Chapagain and Rauniyar, 2020), antipyretic drugs (Fernando et al., 2017; Shafie et al., 2018; Tesfamariam et al., 2019; Chapagain and Rauniyar, 2020), cough and cold preparations (Arrais et al., 2016; Håkonsen et al., 2016; Tesfamariam et al., 2019), anti-allergic/antihistamine drugs (Håkonsen et al., 2016), antacids and anti-reflux drugs (Håkonsen et al., 2016; Tesfamariam et al., 2019), muscle relaxant drugs (Arrais et al., 2016), antimicrobial drugs (Fernando et al., 2017; Aziz et al., 2018; Shafie et al., 2018), and traditional remedies (Shafie et al., 2018). In the above drug categories, analgesics and antibiotics stand out as the most commonly used self-medicated drugs. In our study the majority of participants practiced self-medication no more than twice a month, and usually chose which drugs to buy rather than allowing the seller to make drug recommendations. This phenomenon could also lead to drug-related issues if pharmacists in drug stores do not provide adequate drug-use recommendations.

In addition, the current study discovered that some participants had never met a pharmacist, were unsure whether the seller was a pharmacist, and that they had never received advice from the seller when purchasing drugs in a store. Furthermore, 13.6% were never asked by the seller about their history of drug allergies or pregnancy status. This phenomenon should be noted, as there is a risk of incorrect self-diagnosis, dangerous drug-drug interactions, incorrect administration, incorrect dosage, incorrect choice of therapy, masking of a severe disease, and/or risk of dependence and abuse if people receive insufficient information from healthcare professionals, particularly the pharmacist at the store (Shafie et al., 2018).

In terms of self-medication outcomes, nearly half of the participants reported that taking drugs on their own did not always completely cure their illnesses, and 76.2% sought treatment at a hospital or clinic. In such cases, individuals may incur additional healthcare costs. Although the study captured a smaller number of participants who experienced adverse drug reactions, we discovered that some of those who had adverse drug reactions experienced severe or serious symptoms that disrupted their daily lives or necessitated hospitalization. In such cases, additional healthcare costs may be borne by both individuals and the country.

According to the participants, the most common reasons for self-medication in the current study were the non-seriousness of the illness, easy access to a pharmacy store, the ability to save necessary medications at home, the inconvenience of using health insurance, and the availability of health or drug information on the internet. Several studies have reported different reasons for self-medication, including non-seriousness of the illness (Kassie et al., 2018; Lei et al., 2018; Shafie et al., 2018; Tesfamariam et al., 2019), saving time (Kassie et al., 2018; Lei et al., 2018; Tesfamariam et al., 2019; Chapagain and Rauniyar, 2020), cost effectiveness (Kassie et al., 2018; Lei et al., 2018), ease of accessibility (Tesfamariam et al., 2019; Al-Ghamdi et al., 2020; Chapagain and Rauniyar, 2020) and availability of information on the internet (Lei et al., 2018; Al-Ghamdi et al., 2020).

In terms of people’s basic knowledge about medicines when self-medicating, this study found that the majority of participants are knowledgeable about general medication use. Despite the fact that the majority of study participants had a high level of education, and nearly one-third had a background in the health sciences, we detected some misinformation about antibiotic drugs. More than 20% misunderstood that if diarrhea occurred, antibiotics should be started as soon as possible, and nearly two-thirds were unaware that if a viral infection was confirmed while taking antibiotics, the antibiotics should be stopped. Recent studies have made similar findings on antibiotic knowledge or misuse. In one study, the most common symptoms for which medical students used antibiotics were sore throat, flu, and runny nose (Rathish et al., 2017). Similar to another study, the majority of participants believed that antimicrobials could be used to stop a fever and that antimicrobials were effective in treating common colds (Sambakunsi et al., 2019). Another study found that up to 68% of participants thought antibiotics should be taken for a sore throat (Jamhour et al., 2017). Moreover, a study on antibiotic knowledge and use conducted in Thailand in 2017 revealed that half of the respondents gave the incorrect answer to the statement that antibiotics can cure common cold and flu symptoms; antibiotics can kill viruses; and antibiotics can cause side effects such as diarrhea (Chanvatik et al., 2019). These studies indicated that interventions aimed at increasing antibiotic drug knowledge and/or antibiotic stewardship should be rigorously implemented.

### Limitations

The current study was designed to assess the prevalence of self-medication and the use of some relevant drugs, but it did not include household remedies. So, the prevalence of self-medication may be underestimated. As a result, the findings cannot be applied to all medications. Causality assessment was not run for the reported adverse drug reactions owing to the limitation of the study. As a result, reported adverse drug reactions may not accurately reflect the situation. In terms of assessing participants basic medication knowledge, the high percentages of those answering the knowledge statements may have been overestimated because the investigator did not provide “unsure or I don’t know” responses for each question. Most participants would choose the correct answer simply by guessing not because they are knowledgeable of this statement. Based on the online data collection, the findings of this study may not be representative of the entire population. Furthermore, because participants were asked to provide a history of use and adverse effects, recall bias could affect data accuracy.

## Conclusion

The present study revealed very high prevalence of self-medication. In terms of self-medication practices, the majority of participants chose which drugs to buy rather than allowing the seller to make drug recommendations. NSAIDs, antibiotics, and anti-allergic drugs were the most frequently used medications. The current study discovered that some participants had never met a pharmacist, were unsure whether the seller was a pharmacist, and had never received advice from the seller when purchasing drugs in a store. Furthermore, some participants were never asked by the seller about their history of drug allergies or pregnancy status. In terms of self-medication outcomes, nearly half of the participants reported that taking drugs on their own did not always cure their illnesses completely. As a result, they sought medical attention at a hospital or clinic. A small number of participants experienced adverse drug reactions as a result of self-medication but some had severe symptoms that disrupted their daily lives or required hospitalization. In the current study, the most common reasons for self-medication were the non-seriousness of the illness, easy access to a pharmacy store, the ability to save necessary medications at home, the inconvenience of using health insurance, and the availability of health or drug information on the internet. When examining basic medication knowledge, we discovered that study participants misunderstood some antibiotic drug concepts. According to these findings, more information about the risks of self-medication, drug adverse reactions, antibiotic stewardship, more supervision over the prohibition of over-the-counter drugs and selling practices, and adequate facilities for people's access to medical services should be provided at the policy level.

## Data Availability

The original contributions presented in the study are included in the article/Supplementary Material, further inquiries can be directed to the corresponding author.
